# Use of alternative diluent ACP-Lact® and coconut water powder (ACP) in cryopreservation of goat semen and fixed-time artificial insemination (FTAI) in half-breed goats

**DOI:** 10.1590/1984-3143-AR2023-0081

**Published:** 2023-10-20

**Authors:** Leonardo Alves Rodrigues Cabral, Wallisson Bruno de Morais Pacheco, Suane Silva Alves dos Santos, Adriano da Silva Prado, Luis Alberto Linhares Rufino, Cristiane Clemente de Mello Salgueiro, Ney Rômulo de Oliveira Paula, Assis Rubens Montenegro, José Ferreira Nunes

**Affiliations:** 1 Faculdade de Veterinária,Universidade Estadual do Ceará, Fortaleza, CE, Brasil; 2 Faculdade de Veterinária, Universidade Federal do Piauí, Teresina, PI, Brasil; 3 Centro de Ciências Agrárias, Universidade Federal do Ceará, Fortaleza, CE, Brasil

**Keywords:** goat, alternative diluent, semen freezing, cryoprotection

## Abstract

The aim of the present study was to evaluate semen cryopreservation with ACP-Lact® diluent, which consists of coconut water powder (ACP) added to goat milk powder. After thawing, the samples were evaluated for sperm kinetics, membrane evaluation and *in vivo* insemination. For cryopreservation, a pool was made with the ejaculate of six goats, diluted in four equal aliquots for the respective treatments: T1 (ACP-Lact®); T2 (ACP-Lact® 50%); T3 (ACP + 2.5% egg yolk) and T4 (Tris + 2.5% egg yolk). After dilution of the treatments, the samples were placed in 0.5 ml straws and chilled at a rate of -1.07°C/min. After reaching 4°C and stabilizing for one hour, the straws were placed in nitrogen vapour at -60°C for 15 minutes and then immersed in liquid nitrogen (-196ºC). The straws were thawed in a 37°C water bath and kinetic assessments were performed immediately using a computerized semen analysis program (CSA), viability (EN), membrane functionality (HOST), mitochondrial activity (DAB) and DNA integrity assessment of spermatozoa. For the *in vivo* experiment, ten goats were inseminated, divided into two groups of five goats each, G1 inseminated with ACP-Lact® and G2 with ACP, by fixed-time artificial insemination (FTAI). Regarding the kinetic parameters, the ACP-Lact® treatment showed higher progressive motility (PM) and sperm velocity than the other treatments (36.77%). In the VSL parameter the ACP-Lact diluent was superior to ACP and Tris. In viability the treatment with ACP-Lact® was superior to the treatment with Tris, 95% and 83% respectively. In FTAI two goats were born out of the 5 goats inseminated with ACP-Lact®. It was concluded that the use of ACP-Lact® for cryopreservation of caprine semen is efficient in maintaining seminal parameters during thawing in vitro and in vivo and proved to be a good alternative extender for the caprine species.

## Introduction

Seminal cryopreservation is a biotechnique that allows prolonging the survival of sperm, since sperm cells do not survive for a long time in fresh semen. Its advantages include (a) optimization of the use of breeding stock; (b) transport of genetic material to other regions; (c) possibility of creating semen banks, depending only on a cryogenic cylinder; and (d) possibility of improving herds of several producers (Nunes and Salgueiro, 2011).

This procedure, however, depends on preservation means that provide a favourable environment so that the sperm, when thawed, remains with fertilizing capacity, enabling the final process of artificial insemination and fertilization. According to Nunes and Salgueiro (2011), a good conservation medium must be non-toxic, have a favorable pH and osmolarity similar to that of semen, be easy to prepare and low cost. These characteristics are found in the coconut water-based medium that confers survival and viability to sperm in thawed semen.

Due to the excellent results obtained in the first studies with coconut water in natura, a semen preservation medium based on standardized and stabilized coconut water powder (ACP) was developed, allowing the conservation of its beneficial characteristics, and facilitating its use in regions where the fruit is not available (Nunes and Salgueiro, 2011). ACP has been used in animal reproduction biotechnology, obtaining good results in the conservation of semen from domestic animals such as goats (Oliveira et al., 2011; Brito et al., 2022), sheep (Cavalcante et al., 2014; Brito et al., 2019), pigs (Guimarães et al., 2018) and canids (Uchoa et al., 2012).

Goat semen has the enzyme phospholipase “A” that interacts with membrane phospholipids and catalyzes the hydrolysis of lecithin present in egg yolk, releasing fatty acids and lysolecithins, responsible for the detergent action on plasma membrane lipids, which are highly toxic to sperm (Corteel, 1974); therefore, it is important to search for products that can replace egg yolk; in addition, the use of egg yolk can bring risks to biosafety because it is a favorable medium for contamination, and thus, hinder the commercialization of inseminating doses. In addition, goat semen has a high rate of polyunsaturated fatty acids, making the sperm susceptible to peroxidative damage. Due to these factors, cryopreservation of goat semen with acceptable fertility and quality parameters has proven to be a challenge (Asadpour and Nasrabadi, 2011).

ACP-Lact®, a powdered diluent, which consists of the addition of goat milk powder to ACP diluent, can be a low-cost alternative diluent that can be used without egg yolk, which makes sanitary control more stringent and standardized. In addition, goat's milk has the advantage of being a product with great economic potential for the entire Northeast region of Brazil (Delgado et al., 2020; Oliveira et al., 2022), having higher amounts of medium and short chain fatty acids and smaller fat globules when compared to cow's milk (Delgado et al., 2020), which may represent an advantage in the seminal medium, mainly to avoid a strong interaction with phospholipases present in caprine semen.

The objective of the present study was to evaluate the ACP-Lact® diluent as an alternative for the replacement of the ACP-101c diluent added with egg yolk, in thawed caprine semen, through the parameters of sperm kinetics evaluated in a computerized system (CASA), the conservation capacity of sperm membranes and the fertilizing capacity through fixed-time artificial insemination (FTAI) in fertile mestizo goats.

## Methods

### Location and animal ethics

The coconut water powder (ACP-101c) and the ACP-Lact® product used in the experiment were provided by the company ACP Biotecnologia, incubated at the State University of Ceará. This research was carried out after evaluation and approval by the Ethics Committee on the Use of Animals of the State University of Ceará, Fortaleza, Ceará, Brazil, with protocol no. 2878284/2019. The experiment was carried out at the Laboratory of Goat and Sheep Sperm Technology (LTSCO), inserted in the Integrated Biotechnology Center (NIB) of the State University of Ceará (UECE). The NIB is located in the city of Fortaleza, in the State of Ceará, Brazil, with latitude of 3º43'47” south and longitude of 38º30'37” west and altitude of 16 meters above sea level. The climate of the region, according to the Köppen classification, is hot and humid, with thermal averages ranging from 26 to 27 ºC, maximum of 30 ºC and minimum of 19 ºC.

### Animals and semen collection

Six goats, aged two to four years, with proven fertility were used. The animals were raised under intensive management with feed composed of Tifton hay (Cynodon dactilum) and commercial feed 18% PB, with water and mineral salt at will. Feeding was divided into two parts, with half of the hay and commercial feed offered in the morning and the other half offered in the late afternoon.

Eight ejaculates were collected from six goat sires (n=48) using an artificial vagina specific for small ruminants and a female goat was used as a dummy (Hafez and Hafez, 2004). After collection, the samples were kept in a water bath at 37°C and evaluated for volume and mass motility (0 to 5) (CBRA, 2013). Only ejaculates with volume greater than 0.5ml, mass motility ≥4 and total motility ≥ 80% were used for the experiment. Then, the sperm concentration of each ejaculate (x10^9^ sptz/ml) was determined in a Neubauer chamber (Chemineau et al., 1991).

### Preparation of the diluent

Packets of ACP-Lact® were prepared to be diluted in 50mL of distilled water, so that the final result of the solution has an osmolarity of 300mOsm and a pH of 7.0. The ACP-Lact® packets are also diluted in 50 mL of distilled water, obtaining a pH 7.0 with 300 mOsm.

Distilled water and the antibiotic gentamicin 40mg (Gentatec®, Agro Veterinária) were added to all diluents. They were prepared with the diluent based on coconut water powder specific for the caprine species (ACP-101c) and the ACP-Lact® diluent, which consists of 50% ACP-101 + 50% goat milk powder. A dilution of ACP-Lact® was performed to assess half the concentration, forming ACP-Lact® 50%. ACP-101c was diluted according to the manufacturer's recommendations; 5% egg yolk (O.G.) and 7% glycerol were added to ACP-101 and Tris diluents, while only 7% glycerol was added to ACP-Lact® and ACP-Lact®50% diluents, resulting in the following treatments: T1 (ACP-Lact ®); T2 (ACP-Lact® 50%), T3 (ACP) and T4 (Tris).

### Cryopreservation

The ejaculates were pooled, divided into four aliquots of equal volume and diluted in the respective treatments. After dilution, the samples were placed in 0.5 ml straws, cooled at a rate of -1.07°C/min to 4°C (Bispo, 2005), and kept at this temperature for 60 min (stabilization). After this process, the straws were placed on a freezing ramp, at a distance from the liquid nitrogen (LN) where the temperature remained at -60°C for 15 min, and then immersed in LN and stored in cryopreservation canisters at -196°C.

### Sperm kinetics and membrane evaluation

For the evaluation of sperm kinetics, 500 μL aliquots of the samples were placed in microtubes, diluted in the base diluent (ACP-101c; ACP-Lact® and Tris) to a concentration of 40 x 106 sptz/mL, kept in a water bath at 37 ºC. Subsequently, 10 µL of each diluted sample were analyzed individually in a Makler chamber preheated to 37°C. Images were acquired and processed on a phase-contrast microscope coupled to a digital camera and analyzed on a Computer-assisted Sperm Analysis (CASA) system using the Sperm Class Analyzer® software (SCA®, Microptic SL, Barcelona, Spain) according to the following parameter settings: Contrast - 100; Brightness - 100; Image/second - 24; Optics - Ph+; Camera - makler; Scale - 10x; Particle size - 10 < 70 (µm2); Slow > 10 µm/s; Medium > 45 µm/s; Fast > 75 µm/s; Circular 50% of LIN; Velocity at mid-travel points - 5; Image numbers - 25. The following sperm kinetic parameters were assessed: total motility (TM%); curvilinear velocity (VCL, μm/s); linear velocity (VSL, μm/s) and mean path velocity (VAP, μm/s).

To analyze viability and morphology, smears were made on slides pre-warmed at 37°C with 5 μl of eosin-nigrosin stain (1g eosin, 2g nigrosin, 3.75g sodium citrate and 100ml distilled water q.s.p. 100ml; Baril et al., 1993), added to 5μl of rediluted semen.

Regarding sperm viability, 200 spermatozoa were counted per slide, being considered viable those that did not allow the passage of the dye through the membrane, therefore not stained, and non-viable cells that stained the cytoplasm, therefore stained. For morphology, 200 spermatozoa were counted per slide and primary, secondary and tertiary defects were classified.

For the hypoosmotic analysis, a solution of sodium citrate (0.245g) + fructose (0.45g) in 50ml of distilled water, used in the protocols for the analysis of goat and sheep semen (Oliveira et al., 2013), was prepared. A 10µl aliquot of each treatment was diluted in 100µl of hyposmotic solution and incubated for 1 hour in a water bath at 37°C. After that, each sample was placed in a Makler chamber and analyzed in a computerized system, at 400x magnification, where 200 spermatozoa were counted per sample; and functional membranes were considered those with curled tail and non-functional membranes those with straight tail.

### Assessment of mitochondrial activity (DAB)

The protocol of Hrudka (1987) was performed to assess mitochondrial activity using the 3,3-diaminobenzidine (DAB) test; 0.0045g of DAB was weighed and diluted in 300µl of PBS. A 10µl sample of each treatment was placed in 25µl of DAB solution and incubated for 40 minutes in a 37°C water bath. After incubation, a smear of the solution was made for each treatment and the slides were stored in a cuvette with 10% formaldehyde for 10 minutes. The slides were then washed and left to dry at room temperature.

For analysis, the slides were observed under a phase contrast microscope at 400x magnification. Two hundred spermatozoa were counted/slide and classified according to the degree of staining of the midpiece into four classes class I - spermatozoa with a completely stained midpiece, indicating high mitochondrial activity (DAB I); class II - spermatozoa with more than half of the segments stained (active), indicating medium/medium to high mitochondrial activity (DAB II); class III - spermatozoa with less than half of the segments stained (active), indicating high mitochondrial activity (DAB III); class IV - spermatozoa with a completely unstained midpiece, indicating no mitochondrial activity (DAB IV).

### DNA fragmentation

The percentage of spermatozoa with DNA fragmentation was determined by the Sperm Chromatin Dispersion test (Fernández et al., 2003, 2005). Low molecular weight agarose was boiled for 5 minutes, then a 50 µl aliquot of the agarose was placed in an Eppendorf tube and kept in a 37 °C water bath for 5 minutes. A 25 µl aliquot of the semen sample was added to the Eppendorf containing the agarose. On a pre-prepared slide, 2 µl of semen diluted in the agarose was aliquoted and each spot was duly marked and then covered with slide covers, for a total of eight aliquots per slide. The slides were then placed in a refrigerator (4°C) for 5 minutes. The coverslips were then removed and the slides subjected to a series of solutions: HCL solution (7 min); lysis solution (25 min); distilled water (5 min), and 70%, 90% and 95% alcohol, respectively (2 min each). The slides were then dried at room temperature (22 °C) and then stained with the Panoptic kit. Finally, the slides were washed in distilled water and dried at room temperature (22 °C). Five hundred spermatozoa were evaluated per slide and the percentage of cells with integral DNA (DNAi) was determined by the presence of a large halo of chromatin dispersion around the sperm head and the percentage with fragmented DNA (DNAf) was determined by the absence of the halo.

### Fixed-time artificial insemination and pregnancy assessment

For fixed-time artificial insemination, 10 proven fertile mixed-breed goats, kept in stalls, between 2 and 3 years of age, with an individual score of 2.5 to 3, were used. Intravaginal insertion of medroxyprogesterone acetate (MAP) sponges was performed on day 0. On day 9, 400 I.U. of PMSG (NOVORMON®) and 150 µg of prostaglandin 2α (PGF-2α) were applied intramuscularly (I.M). On day 11, the sponges were removed and 36 hours later artificial insemination was performed. Five goats were inseminated with ACP-Lact® and five with ACP-yolk.

After 32 days a transrectal ultrasound was performed, where it was possible to detect pregnancy in 2 goats of the ACP-Lact® group. After 35 days another ultrasound was performed where it was possible to observe that the pregnancies were still viable. After 152 days of insemination, the goats gave birth.

### Statistical analysis

Data were analyzed using the statistical software R-project© version 3.3.2 (The R Foundation, Vienna, Austria), and submitted to the Shapiro-Wilk normality test and Bartlet's homoscedasticity test. Parametric CASA motility data were subjected to ANOVA followed by the Student-Newman-Keuls test. Parametric data including DNAi, HOST, DAB 1 and DAB 3 were subjected to ANOVA followed by Duncan's test and non-parametric data such as viability, DAB 2 and DAB 4 were subjected to Kruskal-Wallis test and then Dunn's test to compare the means, with a 95% confidence interval. Results were expressed as mean ± standard deviation.

## Results

### Kinetics

The motility parameters such as progressive motility (PM), were higher in the ACP -Lact® treatment, presenting an average of 36.77% of progressive motility after thawing (Table 1), and were significantly higher than the other treatments. In the velocity parameters, in the rapid velocity of spermatozoa, the treatment with ACP-Lact® (T1) was superior to the others (Table2). Regarding the velocity parameters, in the progressive linear velocity parameter (VSL), the ACP-Lact® 50% treatment (T2) was superior to treatments T3 and T4 and similar to ACP-Lact® (T1). In the linearity parameter (LIN), treatment T2 was superior to treatments T3 and T4 and similar to T1 (Table 3). In the parameters of progressive and non-progressive speed there was no significant difference between treatments. However, all treatments showed satisfactory results considering the progressive movement type for thawed semen (Table 4).

**Table 1:** Means ± standard deviation of the sperm parameters total motility (MT), progressive motility (MP), non-progressive motility (NP) and static motility in the treatments ACP-Lact® (T1), ACP-Lact® 50% (T2), ACP (T3) and Tris (T4).

	**T1**	**T2**	**T3**	**T4**
MT	63.54 ± 2.78	56.58 ± 6.20	66.9 ± 7.81	57.33 ± 7.93
MP (%)	36.77a ± 1.4	22.88b ± 4.9	20.1^b^ ± 5	19.13^b^ ± 5.3
NP	31.1 ± 5.7	33.7 ± 3.8	46.8 ± 6	38.2 ± 5.9
Static	32 ± 5	43.3 ± 5.2	33 ± 7.6	42.65 ± 9.9

^a,b^Different superscript letters on the same line (p < 0.05)

**Table 2:** Means ± standard deviation of the fast, médium, slow and static sperm parameters on the treatments ACP-Lact® (T1), ACP-Lact® 50% (T2), ACP (T3) and Tris (T4).

**VELOCITY**	**T1**	**T2**	**T3**	**T4**
Fast	28.38a ± 2.9	17.5b ± 6.4	12.9^b^ ± 4.7	15.1^b^ ± 5.6
Medium	17.6ab ± 0.4	12^b^ ± 4.3	16.4^ab^ ± 5.8	18.8^a^ ± 5.8
Slow	25.5 ± 4.1	27.56 ± 2.9	33 ± 5.6	36.7 ± 4.1
Static	28.4 ± 5.4	46.5 ± 5.9	22.3 ± 9.1	35.1 ± 7.5

^a,b^Different superscript letters on the same line (p < 0.05)

**Table 3:** Means ± standard deviation of the sperm parameters curvilinear (VCL); progressive linear velocity (VSL); médium trajectory velocity (VAP); Linearity (LIN) and Retilinearity (STR) and oscillation index (Wobble) in the treatments ACP-Lact® (T1), ACP-Lact® 50% (T2), ACP (T3) and Tris (T4).

**Speed Types**	**T1**	**T2**	**T3**	**T4**
VCL	68.5 ± 2.33	74.3 ± 3.26	67.16 ± 2.92	66.81 ± 3.34
VSL	27.3^ab^ ± 0.95	30.42^a^ ± 1.57	24.65^b^ ± 1.38	26.45^b^ ± 1.88
VAP	41.9 ± 1.08	44.3 ± 2.19	39.08 ± 1.58	40.16 ± 2.2
LIN	39.93^ab^ ± 1.33	41.52^a^ ± 0.9	36.68^b^ ± 1.12	39.48^ab^ ± 1.48
STR	65.1 ± 1.08	66.78 ± 1.45	62.93 ± 1.39	65.63 ± 1.28
WOBBLE	61.28 ± 1.31	60.92 ± 1.3	58.21 ± 0.65	60 ± 1.37

^a,b^Different superscript letters on the same line (p < 0.05)

**Table 4:** Means ± standard deviation of the sperm parameters fast progressive; slow porgressive and non progressive in the treatments ACP-Lact® (T1), ACP-Lact® 50% (T2), ACP (T3) and Tris (T4).

**WHO**	**T1**	**T2**	**T3**	**T4**
Fast Progressive	13.11 ± 2.7	11.46 ± 3.38	7.71 ± 2.6	7.76 ± 2.4
Slow Progressive	16.15 ± 2.65	14.46 ± 3.0	12.17 ± 4.9	11.7 ± 2.7
Non progressive	38.85 ± 3.5	33.86 ± 4.48	33.73 ± 6.5	40 ± 5.4

(p > 0.05)

### DNA integrity and membrane function (HOST and DAB)

The percentage of DNA integrity (DNAi) was higher in the ACP-Lact® treatment (T1) compared to the others (p<0.5). Treatment T1 stood out with DNAi=90.3%, higher than T2 (ACP-Lact® 50%) and T3 (ACP + egg yolk) (Table 5). Regarding post-thaw sperm viability, T1 (95.2%) was superior to T4 (83.9%) (p<0.05) and similar to T2 and T3 (Table 5).

**Table 5:** Means ± standard deviation of the parameters of DNA integrity (DNAi), reactive membrane (HOST) and goat spermatozoid viability cryopreserved in ACP-Lact® (T1), ACP-Lact® 50% (T2) e ACP (T3) and Tris (T4) post-thawing.

	**T1**	**T2**	**T3**	**T4**
DNAi	90.3±9.4a	82.5 ± 6.3b	85.7 ± 11.6bc	73.6 ± 6.5 ^b^
HOST	67.2±10.9^a^	63.8±7.1^a^	64.9±9.4^a^	55.2±10.9^a^
Viability	95.2±3.4^a^	90.3±3.7^ab^	93.4±3.3ab	83.9±7.3^b^

^a,b^Different superscript letters on the same line (p < 0.05)

In the hyposmotic test, post-thaw sperm results were satisfactory and there was no difference between treatments (p>0.05; Table 5). Similar performance was observed for DAB parameters (Table 6).

**Table 6:** Means ± standard deviation of the parameters mitchondrial activity (DAB) of goat spermatoids cryopreserved in ACP-Lact® (T1), ACP(T2), ACP-Lact® 50% (T3) and Tris (T4) post-thawing.

**DAB**	**T1**	**T2**	**T3**	**T4**
I – High	86.7±6.5	86.2±2.9	92.2±3.2	89.3±4.5
II – Medium	7.2±5.1	3.8±4.2	4.0±2.2	5.3 ± 2.9
III – Low	6.0±4.4	8.5±3.1	3.6±2.1	4.3±2.7
IV - Absent	0.1±0.3	0.5±0.6	0.2±0.5	1.1±0.9

(p>0.05)

### Artificial insemination

Regarding fixed-time artificial insemination (FTAI), the group inseminated with ACP-Lact® had two calvings out of a total of five animals, a pregnancy rate of 40% with cryopreserved semen, but there were no calvings in the group inseminated with ACP. Although the number of animals is small and the data are based only on empirical observation, ACP-Lact® was more efficient in *in vivo* insemination in the sample group used (Table 7).

**Table 7:** Birth rates from Fixed Time Artificial Insemination (FTAI) with sêmen cryopreserved in duluent based on powdered coconut water (ACP) and powdered coconut water added to goat milk (ACP-Lact®) in crossbred goats.

**Animals/Tratament**	**ACP**	**ACP-Lact®**
Goats	5	5
Birth	0	2 females
Tx	-	40%

## Discussion

Several studies have shown a direct relationship between sperm motility and fertilization power (Kjœstad et al., 1993; Bailey et al., 1994; Stålhammar et al., 1994; Januskauskas et al., 2003), which reinforces the importance of this parameter to verify if an animal can be a good breeder. Motility rates in the diluent based on coconut water powder with goat milk powder (ACP-Lact®) and coconut water powder (ACP) were considered satisfactory for insemination, which requires semen with progressive motility of at least 30% (CBRA, 2013).

In fact, Machado et al. (2006), when inseminating sheep using in natura coconut water powder in cooled semen, obtained satisfactory birth rates. Figueiredo et al. (2007) also obtained good birth rates with ram semen cooled for 24 hours in medium containing coconut water. In the study by Brito et al. (2022), coconut water powder with added vegetable oils was used for cryopreservation of goat semen, obtaining good rates of thawed spermatozoa with indexes higher than 30% of total motility, which corroborated with the data obtained in the present experiment.

Goat milk, unlike cow milk, has fats formed largely by medium and short chain fatty acids (Delgado et al., 2020) and fat globules are smaller when compared to those of cow milk. The appearance and types of fat can influence the interaction of the sperm membrane with the diluting medium. Smaller fat globules may interact less with the semen and give more protection against oxidative attack to the sperm cells.

Regarding DNA, sperm with lesions in the nuclear membrane may damage embryonic development and subsequent fetal development (Sakkas and Alvarez, 2010). Chromatin in the sperm nucleus is reported to be vulnerable to oxidative damage, leading to DNA modification and fragmentation (Zribi et al., 2011).

Hong et al. (2010) evaluated the effects of adding vitamin E to the diet on goat semen preservation and detected a negative correlation between progressive motility and rates of sperm with fragmented DNA, and a positive correlation between rates of fragmented DNA and lipid peroxidation in seminal plasma. In the present study, the percentages of intact DNA were satisfactory in all treatments, but the treatment with ACP-Lact® showed better kinetic results, demonstrating greater protective action against nuclear membrane damage.

Goat's milk contains 0.7 mg/kg of average vitamin E concentration (Cruz et al., 2017), in addition, coconut water powder, obtained from fruits with 6 months of maturation, has a concentration of vitamin C that can vary from 19.7 to 94.3 mg/100 ml in its composition (Carvalho et al., 2006), which can provide a natural antioxidant protection factor to the diluent, since it has already been demonstrated that ascorbic acid can recycle degraded vitamin E, raising the antioxidant potential of these micronutrients (Chou et al., 2018) and giving more protection to the DNA membrane.

Oliveira et al. (2013) obtained low correlation between HOST and motility in goat semen. Other authors also obtained a similar coefficient (Martins et al., 2006). The correlation between HOST and other semen characteristics is controversial in different species. Some authors justify this fact by the specificity of HOST, since cellular edema is indicative of the integrity of sperm membrane functions, while motility is dependent not only on the exchange of substances through the membrane, but also on many other biochemical functions, such as sperm metabolism and the microtubular action of fibers in the region of the sperm cause (Oliveira et al., 2013).

The results obtained in the present study showed positive data for the addition of goat milk powder to ACP-101c diluent. The higher percentages of sperm motility, DNA integrity and viability in the *in vitro* tests allowed a high fertilizing potential. The replacement of egg yolk and the use of an alternative bioproduct that provides similar conditions in the cryopreservation of goat semen allowed obtaining better genetic material post-thawing, with greater chances of fertilization and the possibility of raising the fertility rates of the herd. In the present study goats were inseminated proving the *in vivo* efficiency of ACP-Lact®.

In the present study, the DAB parameter showed no difference compared to the other parameters, but the DAB class I results were satisfactory in all treatments. The DAB parameter can be used to evaluate the mitochondrial activity of spermatozoa, but this activity can occur without correlation with motility parameters. Guthrie and Welch (2012) reported that spermatozoa incubated in medium with menadione and hydroperoxide lost 90% of motility but maintained ATP rates. As menadione reacts with hydroperoxide to form superoxide, the authors inferred that the action of reactive oxygen species (ROS) did not affect mitochondria, but interfered with axoneme function, preventing ATP utilization, or interfered with the contractile mechanism. These findings corroborate with the present study, where no significant difference was observed in the DAB parameter between treatments, differing from the kinetic results obtained. Similar results were observed in previous experiments of our laboratory.

The standardization of milk and its powder form allowed the manufacture of a practical bioproduct for use in semen preservation. In this study, despite the insignificant number of females, it was possible to demonstrate that ACP-Lact® can be used for *in vivo* fertilization. New studies, with more tests and parameters, should be carried out to find optimal concentrations and associations that can bring more satisfactory and standardized results.

## Conclusion

In this study, the first to validate ACP-Lact® diluent, it was concluded that the use of ACP-Lact® as an alternative commercial diluent for cryopreservation maintained good post-thaw sperm quality parameters, with increased total motility, progressive motility, viability and DNA integrity, as well as promoting the birth of viable conceptuses.
